# Ischemic Stroke in a Young Patient with Nephrotic Syndrome and Antiphospholipid Syndrome

**DOI:** 10.1155/2020/8828864

**Published:** 2020-11-25

**Authors:** K. K. Neoh, A. S. N. Tang, I. Looi, B. M. Anita

**Affiliations:** ^1^Department of Internal Medicine, Seberang Jaya Hospital, Penang, Malaysia; ^2^Clinical Research Center (CRC), Seberang Jaya Hospital, Penang, Malaysia

## Abstract

We report a case of a 21-year-old man with underlying nephrotic syndrome (NS) secondary to minimal change disease, who developed an ischemic stroke with left hemiparesis. He received intravenous thrombolysis followed by a mechanical thrombectomy. After mechanical thrombectomy, he developed acute kidney injury which subsequently required haemodialysis. Further workup revealed that he had concomitant antiphospholipid syndrome (APS) and NS. He was started on vitamin K antagonist anticoagulant. This case report illustrates the importance of workup in identifying causes of ischemic stroke in a young patient.

## 1. Background

Acute ischemic stroke in young adults are uncommon as compared to the elderly. It accounts for about 10% of all stroke cases. Physical disability caused by stroke in young adults can lead to huge economic loss to patients and their families. In addition, these groups of patients have longer life expectancies at poststroke, which in turn impose greater burden to healthcare systems.

## 2. Case Presentation

We describe a case of a 21-year-old obese Chinese man with underlying nephrotic syndrome (NS) secondary to renal biopsy-proven minimal change disease who suffered from a right middle cerebral artery territory infarct.

He was initially admitted to our hospital six months prior to stroke, with a complaint of generalized body swelling and shortness of breath for one month. Twenty months prior to the weakness of limbs, he received treatment in a private clinic, but subsequently defaulted. Clinical examination revealed anasarca.

His initial workup for NS revealed that his urea was 10.7 mmol/L, serum creatinine was 134 *μ*mol/L, and serum albumin was 7 g/L. His urinalysis results showed protein 4+ and blood 2+. His 24-hour urine protein was 32.3 g/24 hours. Both kidneys were normal in size and echogenic, with preserved cortical thickness on ultrasound. The bipolar length of the right kidney was 10.3 cm and that of the left kidney was 10.4 cm. Ultrasound Doppler of bilateral kidneys showed no evidence of renal vein thrombosis. Autoimmune workup including ANA, Jo-1, La, Ro, U1RNP, Scl, and SM was negative. He had no history of illicit drug abuse or smoking. His hepatitis B, hepatitis C, and HIV serology were negative. Renal biopsy was planned upon improving his edematous state.

He required temporary haemodialysis alternate with ultrafiltration for 6-week duration due to fluid overload and worsening of kidney function (creatinine up to 282 *μ*mol/L and urea of 17.3 mmol/L). He was started on prednisolone 80 mg per day, furosemide 80 mg three times per day, and cyclosporine 25 mg twice per day.

At the second month of treatment, his kidney function improved with the creatinine level of 97 *μ*mol/L and urea of 9.9 mmol/L. He had weight reduction from 120 kg (BMI: 41.5 kg/m^2^) to 100 kg (BMI: 34.6 kg/m^2^). His haemodialysis was withheld. Renal biopsy was performed and was highly suggestive of minimal change disease. His medications were changed to furosemide 40 mg once per day, prednisolone tapering dose to 20 mg once per day, and cyclosporine 50 mg twice per day.

At the fourth month of treatment, his kidney function further deteriorated with the serum creatinine level of 264 *μ*mol/L and urea of 9.0 mmol/L. His albumin was 12 g/L, and 24-hour urine protein was 19.8 g/24 hours. His weight increased to 115 kg (BMI: 39.8 kg/m^2^). The patient was admitted and reassessed. He was not responding to intravenous steroids and diuretics. His immunosuppressive medication was switched from cyclosporin to mycophenolate mofetil (MMF) 500 mg twice per day due to deterioration of renal function.

He visited us in the clinic at the fifth month of treatment, and he was unable to tolerate MMF due to nausea. He was started on tacrolimus 1 g twice per day. His albumin was 10 g/L, serum creatinine was 278 *μ*mol/L, and 24-hour urine protein was 26.4 g/24 hours. Weight remained the same.

At the sixth month of treatment, he presented to the hospital with left hemiparesis. He was afebrile, vital signs were stable, Glasgow Coma Scale of 15/15, blood glucose was 6.0 mmol/L, and NIHSS score of 12 (left facial palsy: 2 marks, left upper limb: 4 marks, left lower limb: 3 marks, left side sensory: 1 mark, dysarthria: 1 mark, and inattention: 1 mark). Muscle power of the left upper limb was graded 0/5 and that of the left lower limb was 2/5, with reduced sensation over the left upper and lower limbs.

Blood investigation revealed: hemoglobin, 8.8 g/dL (normochromic and normocytic); platelet, 580 × 10^3^/*μ*L; WBC, 13.9 × 10^3^/*μ*L; urea, 6.0 mmol/L; creatinine, 316 *μ*mol/L; INR, 1.2; PT, 15.8 sec; and aPTT, 15.8 sec. Venous blood gas revealed metabolic acidosis, with a pH of 7.25 and HCO_3_ of 13.7 mmol/L. Toxicology screening was not done.

**Figure 1:**
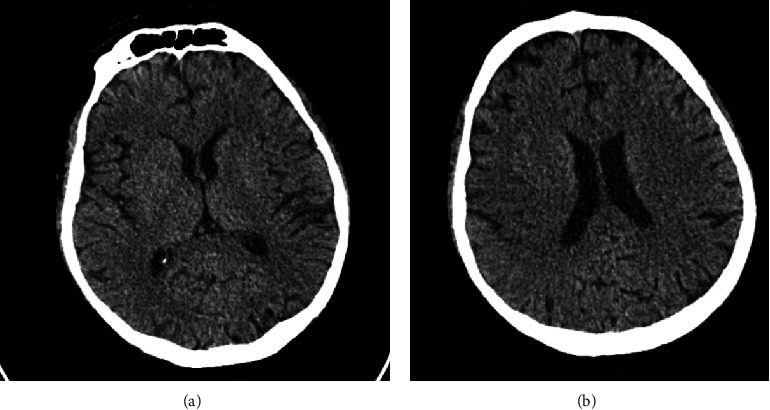
Plain CT brain: normal.

Plain CT brain at 210 minutes poststroke onset showed a normal CT brain (Figure 1), and he was given intravenous thrombolysis with alteplase of a dose of 0.9 mg/kg.

**Figure 2:**
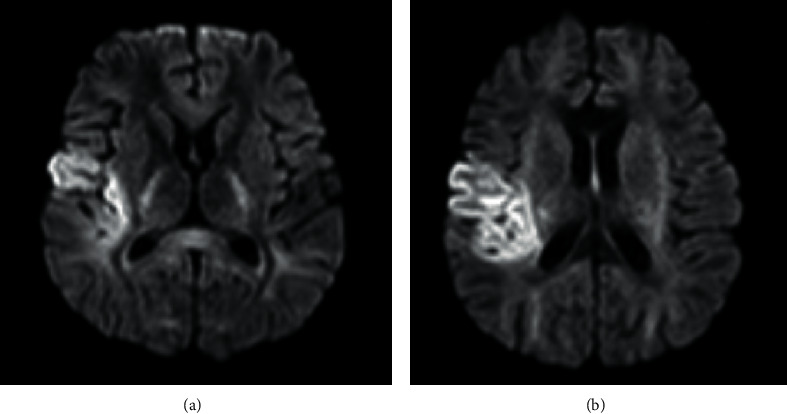
DWI: hyperintensity over right temporal lobe and insular region.

**Figure 3:**
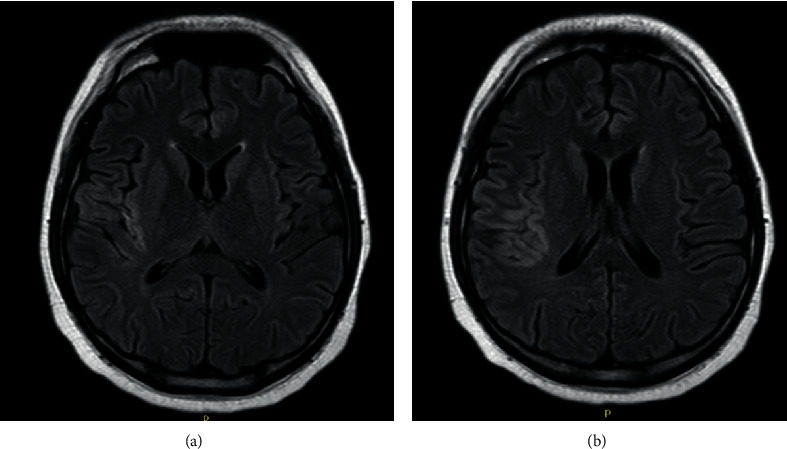
FLAIR: hyperintensity over right temporal lobe and insular region.

**Figure 4:**
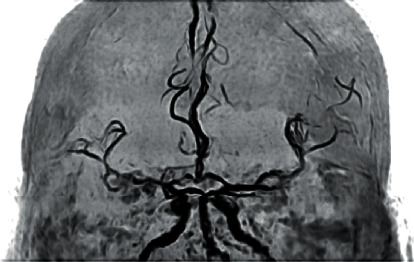
MRA: stenosis at distal M1 segment of the right middle cerebral artery.

MRI and MRA brain scan at 375 minutes poststroke onset and 135 minutes post-rTPA thrombolysis revealed that he had a right middle cerebral artery territory infarct and stenosis at distal M1 segment of the right middle cerebral artery (Figures 2–4). Mechanical thrombectomy was carried out with partial recanalization of M2 segment of the right middle cerebral artery.

His kidney function deteriorated further postcontrast when serum creatinine peaked at 528 *μ*mol/L. He was intubated for respiratory distress as he developed fluid overload. After intubation, he was nursed in intensive care unit and required haemodialysis via a temporary femoral catheter. He also required a tracheostomy to sustain his airway.

He was further evaluated. He had normal C3 and C4 levels, and cANCA and pANCA were negative. Echocardiogram, electrocardiogram, and Doppler ultrasound of carotid arteries were normal. However, his repeated lupus anticoagulant (LA) titre was detected. Hence, the diagnosis of concomitant APS with NS was made. He was subsequently started on vitamin K antagonist (VKA) anticoagulant.

## 3. Outcome and Follow-Up

At three-month poststroke review, he still required regular haemodialysis. It was then decided that he required long-term renal replacement therapy as his kidney function continued to deteriorate.

His Modified Rankin Scale (MRS) after stroke three months was 3. He was able to walk without assistance, but was unable to carry out any other activities. His left upper limb power improved to 2/5, and left lower limb power was 4/5. He further improved at six months after stroke. He still experienced slight disability, but was able to walk independently, and his left upper and lower limbs power improved to 4/5.

## 4. Discussion

Stroke was previously thought as a disease of the elderly, more common in adults aged above 50 years. The incidence of arterial ischemic stroke in young adults less than 45 years of age ranged from 3.4–11.3/100000 people per year, and the common causes were vasculopathy, cardiac defects, pregnancy, hypercoagulable state, smoking, illicit drug use, premature atherosclerosis, young hypertension, low physical activity, and metabolic disease [1].

Ji R's study that recruited 215 young subjects aged between 18–45 years with ischemic stroke or transient ischemic attack showed that the mean age of attack was 37.5 years, and 51% of those diagnosed were males. This group of subjects has a higher incidence rate of traditional vascular risk factors such as hypertension (20%), diabetes mellitus (11%), dyslipidemia (38%), and smoking (34%) [2].

Our patient had NS diagnosed 6 months prior to the onset of stroke, at the age of 20 years. NS was associated with an annual incidence of 9.85% venous thrombosis and 5.5% arterial thrombosis within the first six months of diagnosis [3].

Our patient had proteinuria of 32 g/day, and his albumin level was 0.6 g/dL upon admission. Each 1 g/dL reduction in serum albumin was associated with a 2.13-fold of increased risk of venous thromboembolism, and albumin level of <2.8 g/dL is the most significant independent predictor of venous thrombotic risk [4]. However, the pathogenesis of hypercoagulable state in NS was not well understood, and it was postulated to be due to loss of natural anticoagulants such as antithrombin III, plasminogen, and protein C and S in urine [5].

Arterial thromboembolic event in NS is lesser compared to venous thromboembolism, and there were limited studies addressing this issue. Bakhtawar and colleagues revealed that neither the degree of proteinuria nor serum albumin level was related to arterial thromboembolism [3]. However, sex, age, hypertension, diabetes, smoking, prior history of arterial thromboembolism, and estimated glomerular filtration are parameters of prediction for arterial thromboembolism.

Obesity is another risk factor for stroke in our patient. Obesity-driven chronic inflammation and impaired fibrinolysis are major effector mechanisms of thrombosis in obesity. These mechanisms will trigger endothelial dysfunction, plaque rupture, and platelet activation and end up with arterial thrombosis event [6].

Cyclosporin and tacrolimus are calcineurin inhibitors, and they suppress the immune response by downregulating the transcription of various cytokine genes, particularly interleukin-2 (IL-2), which serves as the major activation factor for T cells. They also have antiproteinuric action through an effect on glomerular permeability [7]. Patients treated with calcineurin inhibitors are at risk of developing acute or chronic kidney injury, and the proposed mechanisms were tubular dysfunction, and rarely thombotic microangiopathy [8]. There were a number of reports on increased risk of thromboembolic events in postrenal transplant patients, and studies suggested that the increase of thromboembolic events was due to multifactorial causes, such as prolonged hospitalization, nephrotic syndrome, proteinuria, steroid usage, and also immunosuppression drugs [9]. Calcineurin inhibitors are associated with a hypercoagulable state in vitro but not in vivo. However, these findings were based on retrospective small sample size studies. More studies are needed to establish the relationship between calcineurin inhibitors and thromboembolic risks [10].

Mycophenolate mofetil (MMF) is an inhibitor of inosine-5′-monophosphate dehydrogenase, and it depletes guanosine nucleotides preferentially in T and B lymphocytes and thus inhibits their proliferation. Studies have shown that MMF is capable of decreasing the in vitro aggregation of platelets in normal subjects [11, 12]. However, the relationship between MMF and thromboembolic events had not yet been established.

APS was diagnosed in our patient where he had positive lupus anticoagulant titre and with a clinical manifestation of arterial thrombosis. APS is an autoimmune multisystem disease with a group of autoantibodies targeting phospholipid binding protein. A critical review of literature done by Androli et al. showed that antiphospholipid frequency was estimated as 6% for pregnancy morbidity, 13.5% for stroke, 11% for myocardial infarct, and 9.5% for deep vein thrombosis [13].

The revised Sapporo diagnosis of APS requires one clinical criteria of vascular thrombosis or pregnancy morbidity and laboratory criteria of presence of either one of the antiphospholipid antibodies (anticardiolipin, anti-beta 2-glycoprotein, or lupus anticoagulant) to be present for at least 2 occasions with 12 weeks apart [14].

APS can lead to kidney damage, either as primary or systemic lupus erythematous- (SLE-) associated APS. Kidney involvement in APS involves a variety of manifestations, such as renal artery thrombosis or stenosis, renal vein thrombosis, allograft loss due to thrombosis after kidney transplantation, and injury to the renal microvasculature [15].

There were limited studies looking into the association of APS and minimal change disease. In Fakhouri et al.'s study of 29 renal biopsies performed in patients with antiphospholipid syndrome, 20 cases showed characteristic features of APS nephropathy, 3 cases were membranous nephropathy, 3 cases were minimal change disease/focal segmental glomerulosclerosis, 2 cases were mesangial C3 nephropathy, and 1 case was pauci-immune crescentic glomerulonephritis [16].

As for the prevention of thromboembolism, anticoagulant (vitamin K antagonist or low-weight molecular heparin) is recommended for all patients with NS who do not have a contraindication.

Antithrombotic medication is generally not recommended for primary thrombosis prevention in patients with APS previously (patients with laboratory criteria but no clinical criteria of APS) as there was lack of high-quality evidence. However, EULAR guideline 2019 recommended low-dose aspirin for asymptomatic APS patients, patients with systemic lupus erythematous (SLE) and APS without prior thrombosis event, nonpregnant woman with a history of obstetric APS, and patients with high title of antiphospholipid antibody [17].

All patients with APS who had a previous thrombotic event are recommended for long-term anticoagulant, either vitamin K antagonist or low-weight molecular heparin [18].

The use of direct oral anticoagulants in NS or APS is controversial, and it is not generally recommended as previous trials were associated with more vascular events compared to that of warfarin, and with limited evidence, further studies are warranted [19–21].

### 4.1. Learning Points

- Young patients with thrombotic events require extensive workup to look for underlying causes of thromboembolism.
- The use of direct oral anticoagulants in NS or APS was not proven well. Current recommendation still favors the use of vitamin K antagonist in these groups.
